# Integrated transcriptomic and proteomic analyses identify the TLR2–CXCR4 axis as a regulator of endothelial cell migration under simulated microgravity

**DOI:** 10.3389/fphys.2025.1701338

**Published:** 2025-12-10

**Authors:** Xiaodong Qin, Ruonan Wang, Chengfei Li, Yikai Pan, Yuan Wang, Xiqing Sun

**Affiliations:** Department of Aerospace Medical Training, School of Aerospace Medicine, Air Force Medical University, Xi’an, China

**Keywords:** simulated microgravity, human umbilical vein endothelial cells (HUVECs), transcriptomics, proteomics, multi-omics integration, TLR2–CXCR4 axis, endothelial cell migration

## Abstract

Simulated microgravity profoundly alters endothelial function, particularly cell migration. However, the mechanosensitive molecular pathways involved remain incompletely understood. In this study, we performed integrated transcriptomic and proteomic analyses of human umbilical vein endothelial cells exposed to simulated microgravity to identify key regulators of endothelial migration. RNA-seq and proteomic profiling identified 964 differentially expressed genes and 183 differentially expressed proteins, primarily enriched in stress response, signal transduction, and angiogenesis pathways. Combined analysis of both datasets revealed four key genes—TLR2, HSPB1, RBM3, and HSPA1B—with more than a twofold change. Protein–protein interaction analysis incorporating 48 endothelial migration—related genes further highlighted TLR2 as a central hub with strong interaction with CXCR4. Functional experiments demonstrated that simulated microgravity significantly enhanced endothelial migration through TLR2 upregulation, while TLR2 activation further promoted this response by increasing CXCR4 expression. These findings identify the TLR2–CXCR4 axis as a previously unrecognized mechanosensitive signaling pathway driving endothelial adaptation to simulated microgravity, offering potential molecular targets for therapeutic intervention against microgravity-induced vascular remodeling.

## 1 Introduction

Extended exposure to microgravity leads to profound cardiovascular deconditioning, characterized by orthostatic intolerance, cardiac atrophy, arrhythmias, and endothelial dysfunction, all of which pose significant risks to astronaut health (Baran et al., 2022). The initial headward fluid shift increases central venous pressure and stroke volume by up to 40%, but subsequent hypovolemia (a 10%–15% reduction in blood volume) and relative anemia compromise cardiac output upon return to normal gravity. Cardiac muscle mass decreases by 10%–20% after long-duration spaceflight, thereby heightening susceptibility to orthostatic hypotension and syncope (Vernice et al., 2020). Moreover, microgravity acts synergistically with space radiation to accelerate oxidative stress, promote vascular remodeling, and impair autonomic regulation, collectively elevating the risk of atherosclerosis and endothelial injury (Patel, 2020). These systemic alterations underscore the urgent need to elucidate the cellular and molecular mechanisms underlying microgravity-induced cardiovascular dysfunction.

Endothelial cells (ECs) are key regulators of vascular homeostasis, modulating vascular tone, permeability, coagulation, and angiogenesis through the mechanosensing of physical forces such as shear stress and hydrostatic pressure (Liu et al., 2013; Charbonier et al., 2019). Under microgravity, the absence of these mechanical cues disrupts endothelial signaling, resulting in alterations in gene expression, protein synthesis, and cellular behavior (Maier et al., 2015; Locatelli and Maier, 2021). Owing to their accessibility and well-established relevance to vascular physiology, human umbilical vein endothelial cells (HUVECs) represent an ideal *in vitro* model for investigating endothelial function (Medina-Leyte et al., 2020; Duranova et al., 2024). Studies have shown that HUVECs exposed to simulated microgravity (MG)—using devices such as the clinostat or random positioning machine—exhibit extensive cellular and molecular alterations (Buravkova et al., 2020; Shi et al., 2012; Kashirina et al., 2021). Collectively, these findings indicate that HUVECs display a complex and multifaceted adaptive response to microgravity, involving diverse signaling pathways.

Transcriptomic studies have revealed that exposure to microgravity induces substantial changes in gene expression within endothelial cells, affecting essential biological functions such as motility, adhesion, and immune regulation. Research on HUVECs under MG conditions has demonstrated alterations in genes associated with cytoskeletal organization, apoptosis, and cell cycle regulation, which collectively contribute to reduced proliferation and increased cell death (Rudimov et al., 2017; Dan et al., 2018). Notably, Buravkova et al. (2017) reported that a 24-h MG environment caused differential expression of 177 genes in endothelial cells derived from umbilical cord blood, many of which were linked to angiogenesis, migration, and cell division—reflecting the cells’ adaptive responses to decreased mechanical load (Rudimov et al., 2017). In addition, transcriptomic investigations have shown that microgravity can modulate endothelial responses to inflammatory stimuli such as lipopolysaccharide (LPS) by reshaping the expression of genes involved in both LPS recognition and cytokine signaling. For instance, Chakraborty et al. (2014) found that microgravity suppressed early immune signaling by downregulating *Lbp*, *MyD88*, and *MD-2*—key genes involved in LPS sensing—while concurrently enhancing the expression of pro-inflammatory cytokines such as IL-6 and IL-8, indicating a reprogrammed inflammatory response under microgravity (Maier et al., 2015).

Recent mass spectrometry–based proteomic studies have begun to elucidate how microgravity (MG) alters the expression of proteins directly involved in endothelial cell migration and vessel formation. For example, Kopp et al. (2021) used a random positioning machine to simulate MG in cultured human umbilical vein endothelial cells for 5 days and subsequently applied label-free quantitative LC–MS/MS to profile protein abundance changes relative to static controls. They identified approximately 120 proteins that changed by ≥ 1.5-fold (p < 0.05), with Gene Ontology enrichment in cytoskeletal organization (e.g., vinculin, talin), extracellular matrix remodeling (fibronectin, laminin), chaperone-mediated proteostasis (HSP70, HSPB1), and focal adhesion assembly (paxillin, FAK). Key findings—including upregulation of HSP70 and vinculin and downregulation of integrin β1—were validated by Western blot, demonstrating that MG induces cytoskeletal disassembly, activates stress-response pathways, and disrupts cell–matrix interactions in endothelial cells (Kashirina et al., 2021). In addition, proteomic analyses of EA. hy926 cells and primary human microvascular endothelial cells cultured on a random positioning machine for 5–7 days revealed differential abundance of cytoskeletal regulators (e.g., vinculin, talin), angiogenic mediators (e.g., angiopoietin 2, PDGF BB), and focal adhesion proteins (e.g., paxillin, FAK), which correlated with impaired formation of three-dimensional tube-like structures (Ma et al., 2014).

 Despite these advances, the molecular mechanisms governing the endothelial response to microgravity remain incompletely understood. To address these gaps, we employed an integrated multi-omics strategy combining transcriptomic and proteomic analyses to comprehensively characterize the complex regulatory networks underlying microgravity-induced endothelial dysfunction (Huang et al., 2025). In this study, we aimed to delineate transcriptomic and proteomic alterations in HUVECs after 48 h of MG exposure. By identifying key differentially expressed genes and proteins, we sought to elucidate the molecular pathways mediating endothelial adaptation to microgravity and to highlight potential therapeutic targets for mitigating vascular dysfunction during spaceflight.

## 2 Materials and methods

### 2.1 Cell culture and experimental conditions

HUVECs were obtained from the American Type Culture Collection (ATCC, Manassas, VA, United States) and maintained in high-glucose Dulbecco’s Modified Eagle’s Medium (DMEM; HyClone, Logan, UT, United States) supplemented with 10% heat-inactivated fetal bovine serum (FBS; HyClone, Logan, UT, United States). The cells were plated at a density of 1 × 10^5^ cells per well on 2.55 × 2.15 cm coverslips within 6-well culture plates and incubated at 37 °C in a humidified environment with 5% CO_2_. To block CXCR4 signaling, we employed the selective antagonist AMD3100 (MedChemExpress, Monmouth Junction, NJ, United States), which was prepared as a 50 µM working solution using sterile ultrapure water.

### 2.2 Simulated microgravity condition

Due to the high cost associated with real spaceflight, most studies investigating the biological effects of microgravity rely on ground-based simulation models. Among these, the clinostat is a widely used and effective tool for simulating microgravity conditions. The 2D-clinostat (2D-RWV, Rotating Wall Vessel), developed by the China Astronaut Research and Training Center (Beijing, China), consists of two rotating components: a horizontal turntable and a vertical turntable. The horizontal chambers rotate around the horizontal axis to minimize the effects of gravity, while the vertical chambers rotate around the vertical axis, serving as rotation controls. After the cells had adhered to the coverslips for 24 h, the coverslips were transferred to culture chambers pre-filled with culture medium. The chambers were divided into two experimental groups: the simulated microgravity (MG) group and the rotation control group (CON). The clinostat was operated at a rotation speed of 30 rpm for 48 h while being maintained in a humidified incubator at 37 °C with 5% CO_2_ throughout the culture period. After 48 h of simulated microgravity exposure, the coverslips were carefully removed from the chambers and processed immediately for subsequent experiments. A schematic representation of the 2D-clinostat setup is provided in Supplementary Figure S1 to illustrate the experimental configuration.

### 2.3 RNA isolation, sequencing and data analysis

After 48 h of culture under either control or rotating conditions, total RNA was extracted from HUVECs using TRIzol reagent (Invitrogen, Carlsbad, CA, United States) following the manufacturer’s protocol. The integrity of the RNA was assessed via electrophoresis on a 1% agarose gel, while its concentration was quantified by measuring absorbance at 260 nm using a UV-2600 spectrophotometer (UNIC, Shanghai, China).

Total RNA was subjected to sequencing using the Illumina HiSeq 2500 platform (Illumina, San Diego, CA, United States), and RNA-seq libraries were prepared accordingly. Briefly, ribosomal RNA was selectively removed to enrich for mRNA, which was subsequently fragmented to the desired length. For each sample, a minimum of 10 μg of total RNA was utilized for library construction following the manufacturer’s protocol. The fragmented RNA underwent sequential ligation of 3′and 5′ adapters, followed by reverse transcription to generate complementary DNA (cDNA). The resulting cDNA was then amplified through 30 cycles of PCR to construct sequencing libraries. Reads with low quality or adapter contamination were filtered out, yielding clean reads for further bioinformatic analysis.

The RNA-seq data analysis was performed as follows: Clean sequencing reads were aligned to the reference genome using HISAT2 (version 2.2.1). The alignment results were subsequently converted into BAM format using SAMtools (version 1.20). Transcript assembly and quantification were conducted using StringTie (version 2.2.3) to generate the gene expression matrix. Differentially expressed genes (DEGs) were identified using DESeq2 (version 1.44.0), with statistical significance defined as *p* < 0.05 and fold change ≥1.5.

GO and KEGG enrichment analyses of differentially expressed genes were performed using the OmicShare tools platform (https://www.omicshare.com/tools). Gene set enrichment analysis (GSEA) was also conducted on the entire gene expression dataset using the same platform using default parameters.

### 2.4 Quantitative real-time PCR

Total RNA was extracted from HUVECs using TRIzol reagent (Invitrogen, Carlsbad, CA, United States) following the manufacturer’s protocol. RNA (500 ng) was reverse transcribed into cDNA using the PrimeScript RT reagent kit (Takara, Shiga, Japan). Quantitative PCR was performed on a LightCycler 480 system (Roche, Basel, Switzerland) with SYBR Premix Ex Taq II (YISHEN, Shanghai, China). The thermal cycling conditions consisted of: 95 °C for 30 s, followed by 40 cycles of 95 °C for 5 s and 60 °C for 30 s. Gene expression was normalized to GAPDH and calculated using the comparative Ct method (2^−ΔΔCT^). The primer sequences used for quantitative RT-PCR are listed in Table 1. Primers were designed and synthesized by Beijing AuGCT DNA-SYN Biotechnology Co., Ltd. (Beijing, China). PCR products were verified by melting curve analysis and agarose gel electrophoresis.

**TABLE 1:** Primers used for each gene.

Gene ID	Forward primer (5′→3′)	Reverse primer (5′→3′)
TLR2	CTTCACTCAGGAGCAGCAAGCA	ACACCAGTGCTGTCCTGTGACA
HSPB1	CTGACGGTCAAGACCAAGGATG	GTGTATTTCCGCGTGAAGCACC
HSPA1B	ACCTTCGACGTGTCCATCCTGA	TCCTCCACGAAGTGGTTCACCA
RBM3	GACCACTTCAGCAGTTTCGGAC	TGGCTCTCATGGCAACTGAAGC
IL6ST	CACCCTGTATCACAGACTGGCA	TTCAGGGCTTCCTGGTCCATCA
BAG3	TGCCAGAAACCACTCAGCCAGA	TGAGGATGAGCAGTCAGAGGCA
CLU	TGCGGATGAAGGACCAGTGTGA	TTTCCTGGTCAACCTCTCAGCG
GAPDH	AGAAGGCTGGGGCTCATTTG	AGGGGCCATCCACAGTCTTC

### 2.5 Protein analysis by LC–MS/MS

#### 2.5.1 Protein extraction

HUVEC samples were subjected to ultrasonic disruption on ice three times using a high-intensity ultrasonic processor (Scientz, Ningbo, China) in lysis buffer containing 8 M urea and 1% protease inhibitor cocktail. The lysates were then centrifuged at 12,000 × g for 10 min at 4 °C to remove insoluble debris. The resulting supernatant was carefully collected, and the protein concentration was measured using a BCA assay kit following the manufacturer’s protocol.

#### 2.5.2 Trypsin digestion

For protein digestion, the sample was first reduced with 5 mM dithiothreitol at 56 °C for 30 min, followed by alkylation with 11 mM iodoacetamide at room temperature for 15 min in the dark. The protein solution was then diluted with 100 mM TEAB to reduce the urea concentration to below 2 M. Trypsin was subsequently added at a trypsin-to-protein mass ratio of 1:50 for an initial overnight digestion, followed by a second digestion at a 1:100 ratio for an additional 4 h. Finally, the resulting peptides were purified using a C18 solid-phase extraction (SPE) column.

#### 2.5.3 4D mass spectrometer

The tryptic peptides were resuspended in solvent A (0.1% formic acid, 2% acetonitrile in water) and directly loaded onto a custom-packed reversed-phase analytical column (25 cm in length, 75/100 μm inner diameter). Peptide separation was performed using a gradient elution, starting with an increase from 6% to 24% solvent B (0.1% formic acid in acetonitrile) over 70 min, followed by a rise to 35% over 14 min, then reaching 80% within 3 min and maintaining this composition for an additional 3 min. The chromatography was conducted at a constant flow rate of 450 nL/min using a nanoElute UHPLC system (Bruker Daltonics, Bremen, Germany).

The eluted peptides were subsequently introduced into a timsTOF Pro mass spectrometer (Bruker Daltonics, Bremen, Germany) via a nano-electrospray ionization (nESI) source. An electrospray voltage of 1.60 kV was applied. Both precursor and fragment ions were detected using a time-of-flight (TOF) analyzer, with an MS/MS scan range of 100–1700 m/z. The instrument operated in parallel accumulation–serial fragmentation (PASEF) mode, selecting precursor ions with charge states between 0 and 5 for fragmentation. Each acquisition cycle included 10 PASEF-MS/MS scans, with a dynamic exclusion time set to 30 s.

#### 2.5.4 Database search

The acquired MS/MS data were analyzed using the MaxQuant search engine (v.1.6.15.0). Tandem mass spectra were matched against the human SwissProt database (20,422 entries), supplemented with a reverse decoy database. Trypsin/P was designated as the protease, allowing for up to two missed cleavages. The mass tolerance for precursor ions was set to 20 ppm in the first search and 5 ppm in the main search, while the fragment ion mass tolerance was set to 0.02 Da. Carbamidomethylation of cysteine was considered a fixed modification, while acetylation at the protein N-terminus and oxidation of methionine were defined as variable modifications. The false discovery rate (FDR) threshold was set to <1%.

### 2.6 Functional analyses and protein-protein interaction network

GO and KEGG annotation was first conducted using eggnog-mapper based on the EggNOG database to assign GO terms related to cellular components, molecular functions, and biological processes (Huerta-Cepas et al., 2019). KEGG pathway annotation was performed by aligning protein sequences to the KEGG database using BLASTP (e-value ≤ 1e-4), with annotations based on the top-scoring hits. Subsequently, enrichment analysis was carried out using Fisher’s exact test, with the entire set of identified proteins as background. Functional categories with a fold enrichment >1.5 or 1.2 and *p* value < 0.05 were considered significantly enriched.

The protein–protein interaction (PPI) network of differentially expressed proteins was constructed using the stringApp plugin in Cytoscape software (version 3.10.3), based on data from the STRING database (https://string-db.org/), with default parameters. The interaction network was visualized and analyzed in Cytoscape. Hub gene analysis was performed using the CytoHubba plugin, and key hub proteins were identified by ranking nodes according to degree centrality.

### 2.7 Western blot

After removal from the rotating chamber, cells on coverslips were rinsed three times with pre-chilled PBS and lysed using RIPA buffer supplemented with 1 mM PMSF. The lysates were collected, vortexed thoroughly, and incubated on ice for 10 min, followed by centrifugation at 12,000 × g for 5 min at 4 °C. Protein concentrations were assessed using the BCA assay and normalized with 5× loading buffer. Equal protein amounts (15 μg per lane) were resolved via SDS-PAGE and subsequently transferred onto methanol-activated PVDF membranes. The membranes were blocked with 5% non-fat milk in TBST for 1 h at room temperature, followed by overnight incubation at 4 °C with the following primary antibodies: anti-TLR2 (1:1000; Cat# 66645-1-Ig, Proteintech, Wuhan, China), anti-HSPB1 (1:1000; Cat# T55934, Abmart, Shanghai, China), anti-HSPA1B (1:2000; Cat# 25405-1-AP, Proteintech, Wuhan, China), anti-RBM3 (1:10000; Cat# 14363-1-AP, Proteintech, Wuhan, China), anti-IL6ST (1:2000; Cat# 67766-1-Ig, Proteintech, Wuhan, China), anti-BAG3 (1:10000; Cat# 83779-4-RR, Proteintech, Wuhan, China), anti-CLU (1:10000; Cat# 84067-4-RR, Proteintech, Wuhan, China), anti-CXCR4 (1:1000; Cat# ab124824, Abcam, Cambridge, United Kingdom), and anti-GAPDH (1:2000; Cat# NC021, Zhuangzhi Biology, Xi’an, China). After thorough washing, membranes were incubated with HRP-conjugated secondary antibodies (1:5000) for 1 h at room temperature. Protein bands were visualized using an enhanced chemiluminescence (ECL) substrate and quantified using ImageJ software (NIH), with GAPDH as the loading control.

### 2.8 Small interfering RNA transfection

HUVECs at 70% confluence were transfected with TLR2 siRNA (sc-35203, Santa Cruz Biotechnology, Dallas, TX, United States) using Lipofectamine 2000 (11668019, Invitrogen, Carlsbad, CA, United States) according to the manufacturer’s protocol. Briefly, cells were washed and maintained in Opti-MEM I Reduced Serum Medium (31985070, Invitrogen, Carlsbad, CA, United States). For each transfection, 50 pmol/mL siRNA and Lipofectamine 2000 (2 μL/mL) were separately diluted in Opti-MEM, incubated for 5 min at room temperature, then combined and incubated for an additional 20 min before adding to cells. Parallel transfections were performed using scrambled siRNA as a negative control. Transfection efficiency was assessed 48 h post-transfection by both qPCR and Western blot analysis. Sequences of the siRNA probes were as follows: NC, 5′-UUCUCCGAACGUGUCACGUTT-3'; siTLR2-1056, 5′-CUGGAUUGUUAGAAUUAGATT-3'; siTLR2-1560, 5′-CUGGAUUGUUAGAAUUAGATT-3'; siTLR2-2480, 5′-CUGCGGAAGAUAAUGAACATT-3'.

### 2.9 Transwell

After exposure to MG for 48 h, HUVECs were collected, digested with trypsin, and resuspended for use in the Transwell migration assay. The Transwell migration assay was assessed using Transwell chambers (Corning Inc., Corning, NY, United States) in a 24-well plate format. Briefly, cells were trypsinized, resuspended in low-serum medium (0.25% FBS), and seeded into the upper chamber (3 × 10^4^ cells/100 μL). The lower chamber contained 600 μL of complete medium (10% FBS) to serve as a chemoattractant. After 12 h of incubation at 37 °C, non-migrated cells were removed from the upper membrane surface with a cotton swab. Migrated cells were fixed with 4% paraformaldehyde, stained with 0.4% crystal violet, and quantified by bright-field microscopy (Nikon).

### 2.10 Statistics

Statistical analyses were performed using GraphPad Prism software (version 8.3.0). Data were expressed as the mean ± SD from three independent experiments. For continuous variables, two-group analyses employed t-tests, whereas comparisons across four and three groups utilized One-way ANOVA supplemented by Dunnett’s *post hoc* analysis and *p* < 0.05 was considered statistical significance.

## 3 Results

### 3.1 Transcriptomic study of endothelial cells under simulated microgravity

**FIGURE 1:**
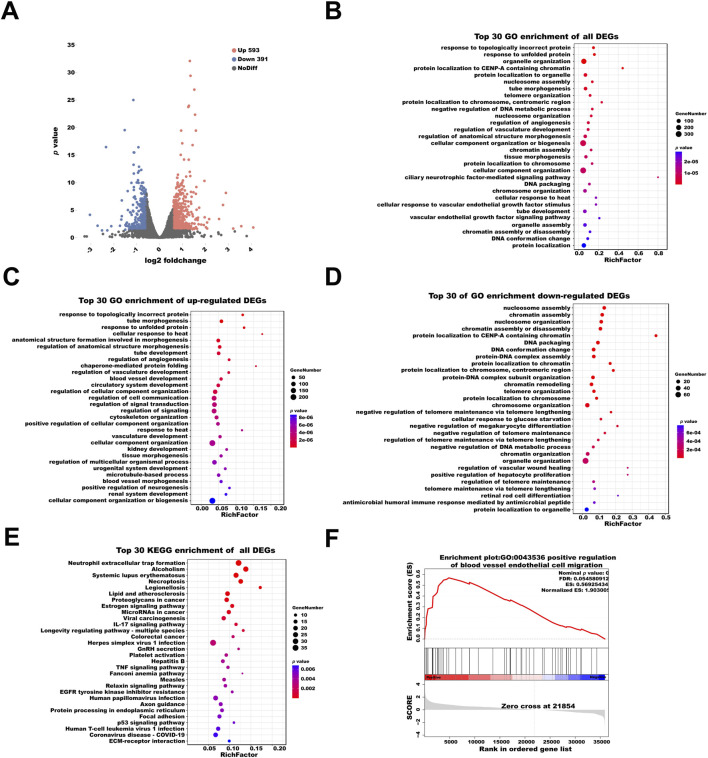
Transcriptomic Analysis of HUVECs under MG. **(A)** Volcano plot displaying differentially expressed genes (DEGs) in HUVECs after 48 h of MG. The x-axis represents log_2_ fold change, and the y-axis represents–log_10_ (*p* value). Red dots indicate significantly upregulated genes, blue dots indicate significantly downregulated genes, and gray dots represent non-significant genes (fold change ≥1.5). **(B)** Gene Ontology (GO) enrichment analysis of all DEGs. The top 30 enriched biological process terms are displayed, with bar lengths representing gene count and color intensity indicating enrichment significance. **(C,D)** GO enrichment analysis of significantly upregulated and downregulated genes, respectively. **(E)** KEGG enrichment analysis of all DEGs. The top 30 pathways are shown. **(F)** Gene Set Enrichment Analysis (GSEA) of the ranked transcriptome based on fold change. The plot displays the enrichment of GO term “positive regulation of blood vessel endothelial cell migration” (GO:0043536). (Statistical significance for all enrichment analyses was defined as adjusted *p* < 0.05).

Through transcriptome sequencing analysis of HUVECs subjected to simulated microgravity (MG) for 48 h, a total of 964 significantly differentially expressed genes (fold change ≥1.5 or ≤0.67, p < 0.05) were identified compared with the control group. Among these, 593 genes were significantly upregulated, while 371 were downregulated (Figure 1A). The complete transcriptome expression matrix and the list of differentially expressed genes (DEGs) are provided in Supplementary Table S1.

GO enrichment analysis of all DEGs revealed significant enrichment in biological processes such as “response to topologically incorrect protein” (GO:0035966) and “response to unfolded protein” (GO:0006986), both indicative of proteostatic stress responses. Additionally, processes such as “tube morphogenesis” (GO:0035239), “regulation of angiogenesis” (GO:0045765) and “regulation of vasculature development” (GO:1901342) were significantly enriched, highlighting their essential roles in endothelial cell migration and angiogenesis (Figure 1B).

Analysis of the upregulated gene set further revealed enrichment in “tube morphogenesis” (GO:0035239) and “response to unfolded protein” (GO:0006986), underscoring their involvement in promoting endothelial migration and adaptation to microgravity-induced stress (Figure 1C). In contrast, the downregulated gene set was enriched in “nucleosome assembly” (GO:0006334) and “chromatin assembly” (GO:0031497), suggesting a potential reduction in transcriptional activity and chromatin remodeling under MG (Figure 1D).

KEGG pathway analysis showed significant enrichment in pathways such as “neutrophil extracellular trap formation” (ko04613), “alcoholism” (ko05034) and “necroptosis” (ko04217). In addition, pathways related to systemic inflammation, such as “systemic lupus erythematosus” (ko05322) were also enriched, supports the notion that MG induces a pro-inflammatory state in HUVECs, which may enhance migratory capacity through the activation of adhesion molecules and chemotactic signaling (Figure 1E).

To further investigate coordinated biological processes beyond individual DEGs, we performed Gene Set Enrichment Analysis (GSEA). The results demonstrated that the gene set associated with “positive regulation of blood vessel endothelial cell migration” (GO:0043536) was positively enriched under MG. The positive normalized enrichment score (NES) indicated that genes within this pathway were predominantly upregulated in the MG group, suggesting transcriptional activation of pro-migratory programs (Figure 1F).

### 3.2 Proteomic study of endothelial cells under simulated microgravity

**FIGURE 2:**
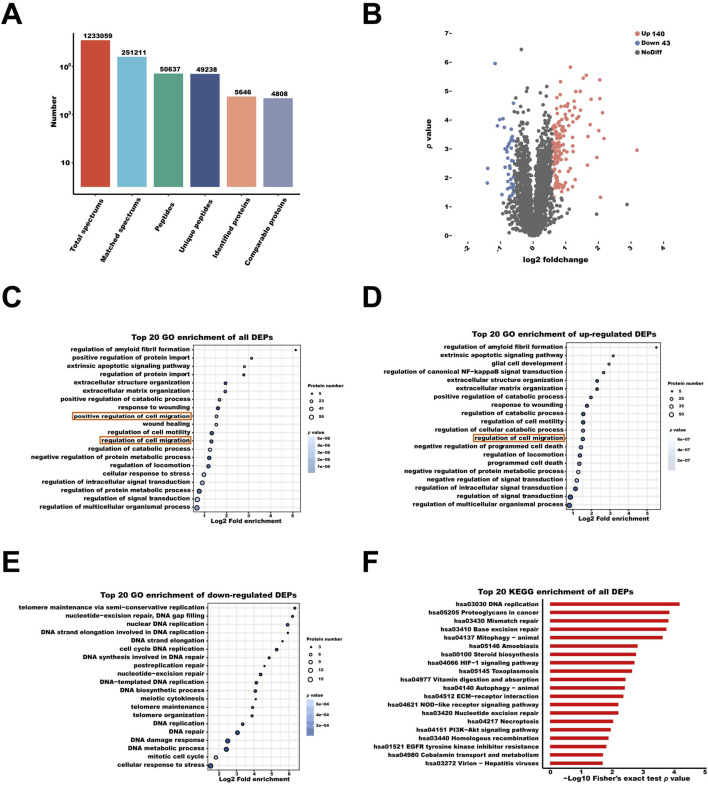
Proteomic Profiling of HUVECs under MG. **(A)** Summary statistics of proteomic analysis using LC MS/MS. **(B)** Volcano plot illustrating differentially expressed proteins (DEPs) after 48 h of MG. The x-axis shows log_2_ fold change, and the y-axis shows–log_10_ (*p* value). Red dots represent significantly upregulated proteins, blue dots indicate significantly downregulated proteins, and gray dots denote non-significant proteins (fold change ≥1.5). **(C)** Gene Ontology (GO) enrichment bubble plot of all DEPs. Bubble size represents the number of proteins enriched in each biological process term, while color intensity indicates enrichment significance. **(D,E)** GO enrichment bubble plot of significantly upregulated and downregulated proteins, respectively. **(F)** KEGG pathway enrichment analysis of all DEPs. The bar graph shows the top 20 significantly enriched pathways. Statistical significance for all enrichment analyses was defined as adjusted *p* < 0.05.

Proteomic profiling of HUVECs following 48 h of simulated microgravity (MG) identified a total of 5,646 proteins, among which 4,808 were quantified with high confidence (Figure 2A). Using a threshold of fold change ≥1.5 and *p* < 0.05, 183 significantly differentially expressed proteins (DEPs) were identified, including 140 upregulated and 43 downregulated proteins (Figure 2B). The label-free quantification (LFQ) intensity values and the complete list of DEPs are provided in Supplementary Table S2.

GO enrichment analysis of all DEPs showed significant overrepresentation of processes such as “regulation of amyloid fibril formation” (GO:1905908), “positive regulation of protein import” (GO:1904591), and “extracellular structure organization” (GO:0043062), implying that MG alters cytoskeletal dynamics and extracellular matrix (ECM) remodeling. Notably, two migration-related GO terms, including “positive regulation of cell migration” (GO:0030335) and “regulation of cell migration” (GO:0030334), were identified and highlighted in orange in Figure 2C. These findings indicate that MG may prime endothelial cells for enhanced motility by reorganizing adhesion and intracellular trafficking systems.

Focusing exclusively on upregulated DEPs, GO enrichment retained strong representation of migration-related pathways, reinforcing the notion that MG preferentially promotes proteomic programs associated with cell motility (Figure 2D). This selective upregulation highlights a cellular shift toward a motile phenotype, possibly as an adaptive mechanism to compensate for the altered mechanical environment.

In contrast, downregulated DEPs were predominantly enriched in nuclear and DNA maintenance processes, including “telomere maintenance via semi-conservative replication” (GO:0032201), “nucleotide-excision repair” (GO:0006289) and “DNA strand elongation” (GO:0022616) (Figure 2E). This downregulation may reflect a transient reduction in proliferation-related activities, favoring the activation of survival and stress-adaptive pathways under MG.

KEGG pathway analysis of all DEPs revealed enrichment in “DNA replication” (hsa03030) “proteoglycans in cancer” (hsa05205), “mismatch repair” (hsa03430), “base excision repair” (hsa03410) and “mitophagy–animal” (hsa04137) (Figure 2F). These enriched pathways reflect broad cellular responses involving DNA maintenance, extracellular matrix organization, and mitochondrial regulation under MG.

### 3.3 Integrated transcriptome and proteome analysis

To comprehensively elucidate the molecular responses of HUVECs under simulated microgravity (MG), we performed an integrated analysis of the transcriptomic and proteomic datasets. To improve transcriptome–proteome comparability and enhance biological insight, we relaxed the protein-level differential expression threshold to ≥1.2-fold in this section. This adjustment enabled the inclusion of a broader set of proteins and increased the number of matched gene–protein pairs, thereby facilitating a more robust and informative multi-omics integration.

**FIGURE 3:**
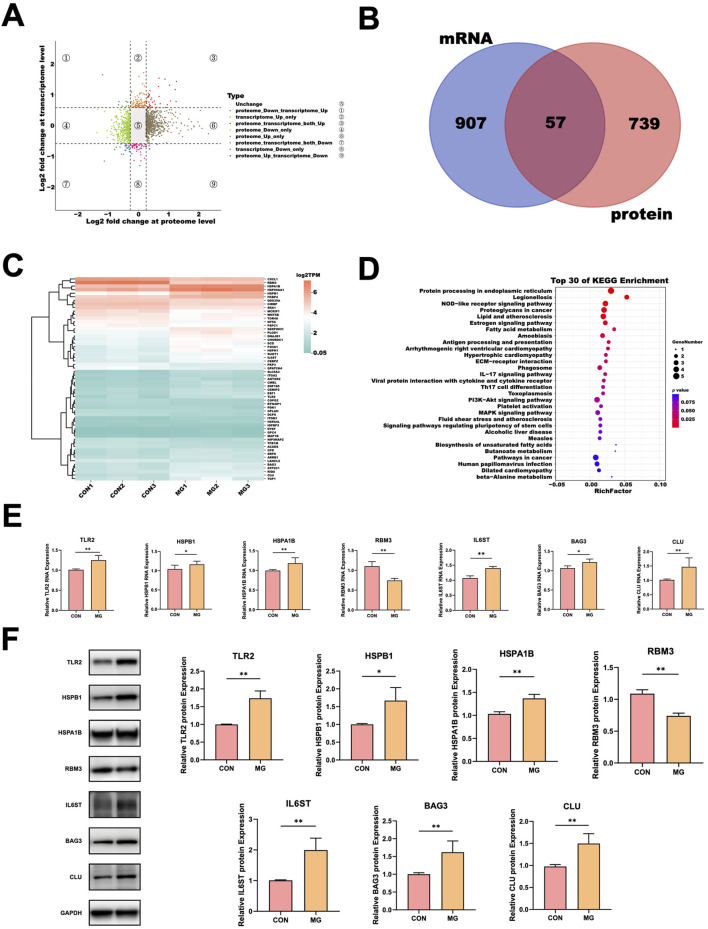
Integrated Transcriptomic and Proteomic Analysis of HUVECs under MG. **(A)** Nine-quadrant diagram of gene and protein expression Changes. Each dot represents a single gene. Different dot colors represent the corresponding expression pattern of genes. The selection thresholds for differentially expressed genes in the transcriptomic and proteomic analyses were set at 1.5-fold and 1.2-fold, respectively, and are indicated by dashed lines. **(B)** Venn diagram showing the overlap of differentially expressed genes identified in the transcriptome and proteome. The transcriptomic DEGs are shown in blue, and the proteomic DEPs are shown in pink. **(C)** Heatmap of the 57 genes differentially expressed in both transcriptomic and proteomic datasets. **(D)** KEGG pathway enrichment analysis for the 57 shared DEGs/DEPs. **(E)** qRT-PCR validation of seven shared differentially expressed genes between transcriptome and proteome datasets, including TLR2, HSPB1, HSPA1B, RBM3, IL6ST, BAG3 and CLU. **(F)** Western blot analysis confirming the protein-level expression of the same seven genes as in panel **(E)**. Representative blots are shown along with quantification relative to GAPDH. All data are presented as mean ± SD (n = 4). Statistical significance was defined as *p < 0.05 and **p < 0.01. CON, control; MG, simulated microgravity.

A nine-quadrant diagram was constructed to illustrate the differential expression patterns of genes and proteins under MG and normal conditions. As shown in Figure 3A, some genes displayed consistent expression trends in both datasets (quadrants 3 and 7), while a few exhibited opposite trends (quadrants 1 and 9). The majority of genes showed significant changes in only one of the two omics datasets (quadrants 2, 4, 6, and 8).

To further identify key regulatory genes involved in the response of HUVECs to MG, we intersected the significantly differentially expressed genes from both the transcriptomic and proteomic analyses. This Venn diagram revealed that 57 genes were significantly differentially expressed in both the transcriptomic and proteomic studies (Figure 3B). Among them, 33 genes were significantly upregulated in both datasets, and 20 genes were significantly downregulated in both datasets. Additionally, 4 genes exhibited opposite expression patterns: WNT5B (Wnt family member 5B) and GPATCH4 (G-patch domain containing 4) were significantly downregulated in the transcriptome but significantly upregulated in the proteome, whereas ZNF185 (zinc finger protein 185) and MAP1B (microtubule associated protein 1B) were significantly upregulated in the transcriptome but significantly downregulated in the proteome. The expression profiles of these 57 genes are illustrated in the heatmap (Figure 3C).

KEGG enrichment analysis of the 57 differentially expressed genes identified in both the transcriptomic and proteomic datasets revealed significant enrichment in several key pathways, including “protein processing in endoplasmic reticulum” (ko04141), “NOD-like receptor signaling pathway” (ko04621), “lipid and atherosclerosis” (ko05417), and “proteoglycans in cancer” (ko05205) (Figure 3D).

Among the overlapping 57 differentially expressed genes, seven genes—toll-like receptor 2 (TLR2), heat shock protein B1 (HSPB1), heat shock protein A1B (HSPA1B), RNA-binding motif protein 3 (RBM3), interleukin 6 signal transducer (IL6ST), Bcl-2–associated athanogene 3 (BAG3), and clusterin (CLU)—were selected for further validation. TLR2, HSPB1, HSPA1B, and RBM3 were chosen because they showed consistent and strong differential expression (fold change >2) at both transcriptomic and proteomic levels, while IL6ST, BAG3, and CLU were included as representative genes involved in cytokine signaling, chaperone-assisted stress response, and extracellular remodeling, respectively. These seven genes were validated by qRT-PCR (Figure 3E) and confirmed at the protein level via Western blot (Figure 3F).

### 3.4 TLR2 promotes endothelial cell migration under simulated microgravity

**FIGURE 4:**
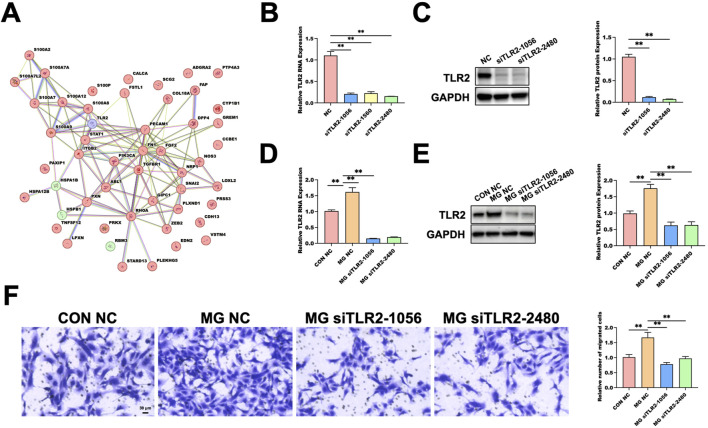
Functional Characterization of TLR2 in Regulating Endothelial Migration under MG. **(A)** Protein–protein interaction (PPI) network integrating four candidate genes (TLR2, HSPB1, HSPA1B, RBM3) with 48 endothelial cell migration–related genes from AmiGO 2. TLR2 is highlighted as a potential hub (purple). **(B)** qRT-PCR validation of TLR2 knockdown in HUVECs after 48 h MG siTLR2-1056 and siTLR2-2480 showed optimal knockdown and were used in subsequent experiments. Data are presented as mean ± SD (n = 3). **(C)** Western blot validation of TLR2 protein knockdown under normal gravity; GAPDH as loading control. Data are presented as mean ± SD (n = 3). **(D)** qRT-PCR validation of TLR2 knockdown under MG (48 h); expression relative to CON NC. Data are presented as mean ± SD (n = 4). **(E)** Western blot analysis of TLR2 under MG following siRNA knockdown; quantified relative to GAPDH. Data are presented as mean ± SD (n = 3). **(F)** Transwell migration assay showing reduced HUVEC migration under MG upon TLR2 knockdown. Data are presented as mean ± SD (n = 3). Statistical significance was defined as **p < 0.01. MG, MG group; CON, control; NC, negative control.

To explore the underlying molecular mechanisms, we performed an integrated analysis of transcriptomic and proteomic datasets in the previous section. We focus on four genes–TLR2, HSPB1, RBM3, and HSPA1B–with fold changes greater than 2 in both omics layers in this section to further investigate their potential roles in endothelial cell responses under simulated microgravity (MG). In parallel, 48 genes annotated with “endothelial cell migration” were retrieved from the AmiGO 2 database to construct an extended gene set relevant to endothelial motility. Subsequently, a protein–protein interaction (PPI) network was established by combining the four candidate genes with the 48 migration-associated genes. Using the STRING database, we generated a high-confidence network to visualize potential interactions among proteins involved in endothelial migration. The PPI analysis revealed that TLR2 exhibited multiple interactions within the network (Figure 4A). To quantitatively assess gene importance, we conducted a hub gene analysis based on network centrality metrics. TLR2 ranked the highest among the four candidates, suggesting its central regulatory position in response to MG.

To validate the function of TLR2, we performed siRNA-mediated knockdown and identified two siTLR2 constructs (siTLR2-1056 and siTLR2-2480) that achieved the most effective suppression, as confirmed by qRT-PCR and Western blot (Figures 4B,C). These two siRNAs were used for subsequent functional assays. To further assess the involvement of TLR2 under MG, siRNA transfection was performed prior to clinorotation. The knockdown efficiency of siTLR2 under MG was verified at both the mRNA and protein levels (Figures 4D,E). Transwell assays showed that the number of migrated cells in the MG NC group was approximately 1.7-fold higher than in the CON NC group, whereas TLR2 silencing significantly reduced this MG-induced enhancement (Figure 4F). These data demonstrate that TLR2 is required for the promotion of endothelial cell migration under MG.

### 3.5 The TLR2-CXCR4 axis facilitates endothelial cell migration under simulated microgravity

**FIGURE 5:**
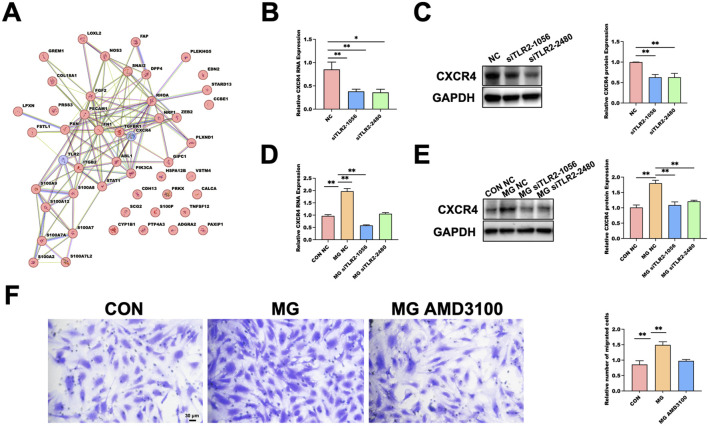
Functional Validation of the TLR2–CXCR4 Signaling Axis as a Mediator of Microgravity-Induced Endothelial Migration. **(A)** Protein–protein interaction (PPI) network constructed with TLR2 and CXCR4 together with 48 endothelial migration–related genes retrieved from the AmiGO 2 database. **(B)** qRT-PCR showing reduced CXCR4 mRNA expression in HUVECs following TLR2 knockdown under normal gravity. Data are presented as mean ± SD (n = 3). **(C)** Western blot validation of CXCR4 protein reduction in response to TLR2 knockdown under normal gravity. GAPDH serves as the loading control. Data are presented as mean ± SD (n = 3). **(D)** qRT-PCR showing that TLR2 knockdown decreases CXCR4 mRNA expression under 48 h MG. Data are presented as mean ± SD (n = 4). **(E)** Western blot analysis confirming reduced CXCR4 protein expression under MG after TLR2 knockdown. GAPDH used as loading control. Data are presented as mean ± SD (n = 3). **(F)** Transwell assay demonstrating that pharmacological inhibition of CXCR4 by AMD3100 attenuates MG-enhanced HUVEC migration. Data are presented as mean ± SD (n = 3). Statistical significance was defined as *p < 0.05 or **p < 0.01. MG, MG group; CON, control; NC, negative control.

Our previous experiments demonstrated that regulation of C-X-C Motif Chemokine Receptor 4 (CXCR4) contributes to enhanced HUVECs migration under simulated microgravity (MG) (Ohori et al., 2021). To further investigate the potential molecular link between TLR2 and CXCR4, we performed an integrative analysis focusing on their possible interaction. In addition to these two targets, we retrieved 48 genes annotated with the biological process “endothelial cell migration” from the AmiGO 2 database and combined them with TLR2 and CXCR4 to construct a protein–protein interaction (PPI) network using the STRING database (Figure 5A). The network analysis revealed an interaction between TLR2 and CXCR4. Subsequent hub gene analysis showed that CXCR4 exhibited a high degree of centrality within the network, suggesting its key role in regulating endothelial migration–related processes under MG.

Under normal gravity conditions, TLR2 silencing significantly reduced CXCR4 expression at both the mRNA (Figure 5B) and protein (Figure 5C) levels, as determined by qRT-PCR and Western blot analysis. After 48 h of MG exposure, TLR2 knockdown similarly led to a marked decrease in CXCR4 mRNA (Figure 5D) and protein expression (Figure 5E). Furthermore, treatment with AMD3100, a selective CXCR4 antagonist, during clinorotation significantly suppressed the MG-induced enhancement of HUVEC migration, as evidenced by Transwell assays (Figure 5F). These results indicate that TLR2 regulates CXCR4 expression and that inhibition of either component diminishes the pro-migratory effects observed under MG, supporting the existence of a TLR2–CXCR4–dependent mechanism mediating endothelial migration.

## 4 Discussion

Simulated microgravity (MG) disrupts cardiovascular homeostasis by altering biomechanical signals in vascular tissues, including fluid distribution and shear forces, which impair endothelial function. As mechanosensitive cells, endothelial cells transduce these changes into biochemical signals regulating vessel tone, remodeling, and permeability. Our findings align with prior studies showing MG induces oxidative stress (Versari et al., 2013), apoptosis (Li et al., 2019), and cytoskeletal alterations (Maier et al., 2015), predisposing the system to deconditioning. For instance, Pan et al. (2025) linked MG to apoptosis via post-translational modifications impairing mitochondrial function (Pan et al., 2020), while Kang et al. (2011) reported downregulation of PI3K/Akt signaling (Kang et al., 2011). Wang et al. (2024) further highlighted upregulation of mechanosensitive channels like those mediating migration. Additionally, MG perturbs extracellular matrix remodeling (Buravkova et al., 2021; Martinez et al., 2024) and receptor signaling, with TLR2 implicated in inflammatory responses exacerbating endothelial stress (Jiang et al., 2011). These observations underscore MG’s multifaceted impact on endothelial integrity, central to vascular stability.

Transcriptomic profiling of HUVECs under 48-h MG revealed 964 differentially expressed genes (593 upregulated, 371 downregulated), providing insights into cellular adaptations. This pattern reflects stress-induced responses in endothelial cells, consistent with Ohori et al. (2021), who identified networks in senescence involving stress, splicing, and cytoskeletal genes (Ohori et al., 2021). Li et al. (2023) reported overlapping oxidative stress-responsive genes (Li et al., 2023), while Abdelgawad et al. (2021) noted subpopulation heterogeneity in single-cell analyses (Abdelgawad et al., 2021). Our data emphasize enhanced cell–cell interactions and integrin signaling, echoing Afshar et al. (2023) on inflammatory and apoptotic gene induction (Afshar et al., 2023). Fu et al. (2019) documented MG-specific signatures, including non-coding RNAs (Fu et al., 2020). This transcriptomic fingerprint validates MG’s unique effects and positions it as a potential biomarker for endothelial dysfunction, informing future interventions.

Proteomic analysis identified 5,646 proteins (4,808 quantifiable), with 183 differentially expressed (140 upregulated, 43 downregulated) under MG. These changes complement transcriptomic shifts, highlighting post-transcriptional regulation in stress response, receptor signaling, and transduction pathways. Mehlferber et al. (2022) revealed isoform diversity in HUVECs (Mehlferber et al., 2022), while Yi et al. (2020) described metabolic and proteomic alterations in senescence (Yi et al., 2020). Tang et al. (2023) linked epigenetic regulation to angiogenic proteins (Tang et al., 2023). Our results show cytoskeletal and adhesion complex shifts, aligning with Donald et al. (2024) and Wang et al. (2023) on mechanical stress effects (Ingber, 2002; Wang et al., 2001). Integrating proteomics with transcriptomics thus offers a fuller view of MG-induced adaptations at mRNA and protein levels.

Multi-omics integration, visualized via a nine-quadrant diagram, captured concordant and discrepant changes between transcriptomic and proteomic layers in MG-exposed HUVECs. This strategy enhances mechanistic insights and target identification beyond single-omics analyses, advancing precision cardiovascular research. This approach, identifying 57 overlapping differentially expressed genes/proteins, reveals pivotal nodes in endothelial adaptation. Among the genes with significant differential expression in both omics layers in this study, TLR2 has been shown to play a significant role in promoting cell migration. Additionally, TLR2 signaling has been implicated in modulating leukocyte migration during tissue injury (Yao et al., 2013; Khandoga et al., 2009). HSPB1, also known as HSP27, is a small heat shock protein that regulates actin cytoskeletal dynamics and cell motility. Phosphorylation of HSPB1 enhances its recruitment to actin filaments, promoting cytoskeletal remodeling and migration (Lam et al., 2022; Doshi et al., 2010). HSPA1B, another heat-shock family member, supports protein folding and stress response, promoting cellular survival and potentially aiding migration under stress (Haase and Fitze, 2016; Xie et al., 2019). RBM3 acts as an RNA chaperone, stabilizing VEGF mRNA to facilitate angiogenesis and directional migration (Pilotte et al., 2018). IL6ST is the common signal‐transducing subunit of IL-6 family receptors; its activation of JAK/STAT3 signaling enhances endothelial proliferation and chemotactic movement during vascular repair (Zegeye et al., 2018). BAG3 serves as a co-chaperone in selective autophagy, regulating focal adhesion turnover and increasing HUVEC migratory capacity under stress (Diao et al., 2022). CLU is a secreted glycoprotein that protects endothelial cells from oxidative injury and stimulates migration by modulating matrix metalloproteinases and adhesion molecules (Trougakos, 2013; Du et al., 2025). In addition, Lin et al. (2025) used machine learning for similar integrations in cardiovascular risk prediction (Lin et al., 2025), while databases like CVD Atlas facilitate clinical correlations (Qian et al., 2025). Alemu et al. (2025) emphasized gene-environment interactions (Alemu et al., 2025), and multi-omics resolves mRNA-protein paradoxes (Vogel and Marcotte, 2012; Aviner et al., 2015).

A key result is the validation of the TLR2–CXCR4 axis mediating HUVEC migration under MG. TLR2, beyond innate immunity, regulates cardiovascular migration and cytoskeletal dynamics. Our datasets showed TLR2 upregulation after 48-h MG, with knockdown reducing CXCR4 levels and migration. This causal link resonates with Bezhaeva et al. (2022) on TLR’s reparative roles (Bezhaeva et al., 2022), and Meteva et al. (2023) on matrix interactions enhancing migration (Meteva et al., 2023). Wang et al. (2024) linked MG to CXCR4 upregulation (Wang et al., 2024), while Colleselli et al. (Colleselli et al., 2023) and Wilhelmsen et al. (Wilhelmsen et al., 2012) described TLR2 pathways converging on adhesion and chemotaxis. AMD3100 (CXCR4 antagonist) attenuated MG-enhanced migration, aligning with Miao et al. (2020) in vascular remodeling models (Miao et al., 2020). Targeting this axis holds therapeutic promise for MG-related cardiovascular issues.

## 5 Conclusion

This multi-omics study under simulated microgravity (MG) provides critical data on endothelial cell adaptations, supporting research on cardiovascular deconditioning associated with MG. In addition, this study reveals that MG activates the TLR2/CXCR4 signaling axis to potently enhance endothelial cell migration. Our integrated transcriptomic and proteomic analyses not only map the molecular responses of HUVECs under MG but also lay a data-driven foundation for understanding vascular deconditioning in spaceflight.

## 6 Limitations

Several limitations of this study warrant consideration. First, while our 2D clinostat effectively simulated microgravity at 30 rpm, achieving a residual gravity of ∼10^–3^ g, we did not perform a dedicated sensitivity analysis of rotation speed. Although this speed is standardized in the literature for minimizing centrifugal artifacts and shear stress, variations could subtly influence outcomes, and future optimizations may refine the model.

Second, the 48-h exposure period, constrained by clinostat oxygenation limits (maximum continuous rotation ∼72 h), captures acute-to-mid-term molecular adaptations but may not fully recapitulate chronic responses observed in long-term spaceflight (weeks to months). Incorporating multiple time points (6–48 h) mitigated this to some extent; however, advanced simulators or space-based experiments are needed to elucidate sustained remodeling.

Finally, although our knockdown experiments establish a causal relationship between TLR2 and CXCR4, with TLR2 acting upstream to regulate CXCR4 expression, the precise molecular mechanism in this model remains to be fully elucidated. Existing literature indicates that TLR2, a membrane-bound pattern recognition receptor, likely modulates CXCR4 via canonical downstream pathways, such as the MyD88-dependent NF-κB cascade or PI3K/Akt signaling, which can influence transcriptional or post-transcriptional regulation of chemokine receptors like CXCR4. For instance, in infection models, TLR2 facilitates CXCR4 expression and function through physical co-association and cross-talk, enhancing downstream signaling that promotes migration and inflammation. TLR2-CXCR4 interactions may also involve β-arrestin2-mediated pathways, regulating endocytosis and signaling to indirectly affect CXCR4 levels. In bacterial invasion contexts, TLR2 exploits CXCR4 to inhibit MyD88-dependent antibacterial responses, suggesting post-transcriptional or trafficking-based regulation. Furthermore, intervention with the CXCR4 antagonist AMD3100 significantly attenuated MG-enhanced HUVEC migration, substantiating CXCR4’s functional role. These observations align with Miao et al. (2020), who reported analogous effects in models of cell migration and vascular remodeling, thereby underscoring the therapeutic potential of targeting the TLR2–CXCR4 axis in cardiovascular diseases.

Future studies could employ specific inhibitors (e.g., for MyD88, NF-κB, or PI3K/Akt) or co-immunoprecipitation assays to directly assess TLR2-CXCR4 interactions and downstream signaling, thereby clarifying the regulatory axis and building upon our findings.

## Data Availability

The raw RNA-sequencing data generated in this study have been deposited in the Genome Sequence Archive for Human (GSA-Human) of the National Genomics Data Center, China National Center for Bioinformation/Beijing Institute of Genomics, Chinese Academy of Sciences. The dataset is publicly accessible under the accession number HRA015094 at the following URL: https://ngdc.cncb.ac.cn/gsa-human.
