# Supplementary material for: Integrated transcriptomic and proteomic analyses identify the TLR2–CXCR4 axis as a regulator of endothelial cell migration under simulated microgravity
Source: Front Physiol. 2025 Dec 10;16:1701338. doi: 10.3389/fphys.2025.1701338 (PMC12727572; doi:10.3389/fphys.2025.1701338)
Supplement: Supplementary file 2 [file DataSheet1.pdf]

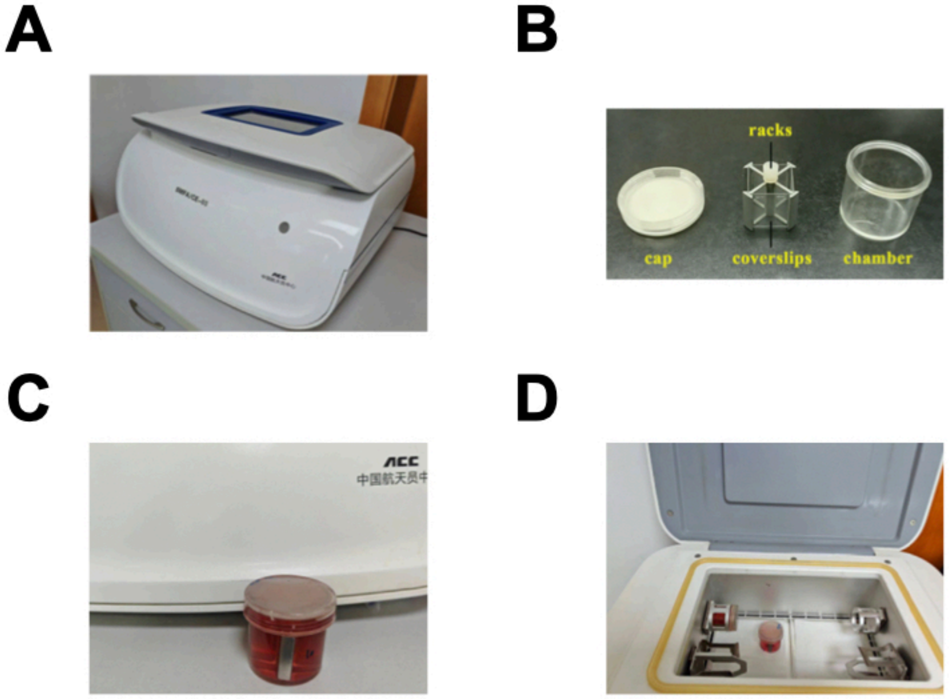

Figure 1. A schematic representation of the 2D-clinostat setup. (A) The two-dimensional (2D) clinostat used in this study was specifically developed by the China Astronaut Research and Training Center to simulate the effects of microgravity by continuously rotating cell cultures around a horizontal axis, thereby minimizing the net gravity vector over time.

(B) Human umbilical vein endothelial cells (HUVECs) were cultured on coverslips placed within sealed chambers completely filled with culture medium. Each chamber contained four coverslips mounted in custom-designed rackets to ensure stable positioning and prevent movement during rotation.

(C, D) To minimize shear stress, the chambers were carefully filled to eliminate air bubbles and hermetically sealed prior to rotation. These precautions ensured that the observed cellular responses were attributable to simulated microgravity rather than fluid flow–induced shear forces.
